# Advances in Endoscopic Management of Endobronchial Carcinoid

**DOI:** 10.3390/jcm12165337

**Published:** 2023-08-16

**Authors:** Gaetana Messina, Davide Gerardo Pica, Giuseppe Vicario, Mary Bove, Giovanni Natale, Vincenzo Di Filippo, Francesca Capasso, Rosa Mirra, Francesco Panini D’Alba, Giovanni Conzo, Tecla Della Posta, Noemi Maria Giorgiano, Giovanni Vicidomini, Damiano Capaccio, Valentina Peritore, Leonardo Teodonio, Claudio Andreetti, Erino Angelo Rendina, Alfonso Fiorelli

**Affiliations:** 1 Thoracic Surgery Unit, University of Campania “Luigi Vanvitelli”, 80131 Naples, Italy; davide_pica@hotmail.it (D.G.P.); vicariogiuseppe@outlook.it (G.V.); bovemary10@gmail.com (M.B.); dott.natale.giovanni@gmail.com (G.N.); francesca.capasso93@gmail.com (F.C.); rosamirra92@yahoo.it (R.M.); franzp@fastwebnet.it (F.P.D.); mednoe@outlook.it (N.M.G.); giovanni.vicidomini@unicampania.it (G.V.); fiorelli.alfonso@gmail.com (A.F.); 2 Department of Traslational Medical Sciences, University of Campania “Luigi Vanvitelli”, 80131 Naples, Italy; giovanni.conzo@unicampania.it (G.C.);; 3 Operative Unit of Endoscopy and Respiratory Pathophysiology, “Maria Santissima Addolorata” Hospital, 84025 Eboli, Italy; damiano.capaccio@aslsa2.it; 4 Thoracic Surgery Unit, Sant’Andrea Hospital, La Sapienza—Università di Roma, 00189 Roma, Italy; valentina.peritore@uniroma1.it (V.P.); leonardo.teodonio@uniroma1.it (L.T.); claudioandreetti@libero.it (C.A.); erinoangelo.rendina@uniroma1.it (E.A.R.)

**Keywords:** bronchial carcinoid, endobronchial treatment, argon plasma coagulation, rigid broncoscopy

## Abstract

Introduction: Bronchial carcinoid (BC) tumors represent between 1% and 5% of all lung cancers and about 20–30% of carcinoid tumors; they are classified into two groups: typical and atypical bronchial carcinoids. The aim of the present study was to review the results of endoscopic treatments as an alternative to surgical treatment in selected patients. Materials and methods: The present study was a retrospective and multicentric study, in which all data were reviewed for patients with BC in the central airways, referred to the Thoracic Surgery Units of Luigi Vanvitelli University of Naples and Sant’Andrea Hospital in Rome between October 2012 and December 2022 Overall, 35 patients, 13 of whom were female, were included in the study (median age, 53 years; range, 29–75 years). All patients underwent rigid bronchoscopy combined with flexible bronchoscopy. Tumor clearance was mostly performed by use of Argon Plasma Coagulation or Thulep Laser, mechanical debridement and excision with the use of forceps and aspirator through the working channel of the 8.5 mm-sized rigid bronchoscope. There were no complications during the treatment. Results: Endobronchial treatment provided complete tumor eradication in all patients; two patients had controlled bleeding complications; however, bleeding was well controlled without patient desaturation, and only one patient died of renal failure during the follow-up period. We found two recurrences in the left and right main bronchus, in patients with atypical carcinoma during fiberoptic bronchoscopy follow-up. Only one patient died of renal failure. At the first analysis, there were no significant differences between the patients receiving endobronchial treatment and patients receiving surgical treatment in the present study (*p*-value > 0.05—it means statistically insignificant). Conclusions: Endobronchial treatment is a valid and effective alternative for patients with BC unsuitable for surgery.

## 1. Introduction

Bronchial carcinoids (CB) are classified as neuroendocrine neoplasms. Neuroendocrine neoplasms (NENs) are a heterogeneous group of epithelial neoplastic lesions [1]; they derive from specialized cells, producers of amines and peptides dispersed throughout the diffuse endocrine system [2].

These neoplasms occur most frequently in the gastrointestinal tract (48%), pancreas (9%) and lungs (25%), but can also develop in other organs, including the prostate, breast, skin and thymus [3]. CB are rare neoplasms, constituting 12–25% of all carcinoids and 2–5% of all broncho-pulmonary tumors with a mean age of incidence of 50–60 years and an annual incidence of 2.3–2.8 cases/million inhabitants/year [4,5].

Histologically, they arise in the bronchial and bronchiolar epithelium and their origin cell type is either Kulchitsky cells (neuroendocrine cells or argentaffin or Feyrter or APUD system, from “amine precursor and uptake decarboxylation”) located in the basal layer of the bronchial lining epithelium and in the bronchial mucosa (between the basement membrane and the columnar epithelium) mainly at the level of the bifurcation of the lobar bronchi.

They are classified into two groups: typical and atypical bronchial carcinoids, according to the 1999 World Health Organization classification which accepted the criteria proposed by TRAVIS based on the correlation between histologic differences and clinical prognosis of the patients [6]. Typical bronchial carcinoids (<2 mitoses/2 mm^2^ of viable tumor) have a good prognosis with a 10-year survival rate of 90% compared with atypical carcinoids (2 to 10 mitoses/2 mm^2^, necrosis or architectural disruption) which have a higher rate of metastasis and recurrence, with a 10-year survival of less than 60% [6]. 

The diagnosis of endo-bronchial carcinoid is based on a bronchoscopic biopsy. However, due to the hemorrhagic nature of the tumor, the number of specimens obtained is often small, making it difficult to obtain an accurate diagnosis with flexible bronchoscopy alone. The use of rigid bronchoscopy can not only help in obtaining a diagnosis, but also be helpful in treatment planning. 

The treatment of bronchial carcinoid consists of surgical removal with or without adjuvant chemotherapy and/or radiation therapy. The aim of the present study was to review the results of endoscopic treatments as an alternative to surgical treatment in selected patients.

## 2. Materials and Methods

The present study was a retrospective and multicentric study, in which all data were reviewed for patients with BC in the central airways, referred to the Thoracic Surgery Units of Luigi Vanvitelli University of Naples and Sant’Andrea Hospital in Rome between October 2012 and December 2022.

Patients and family members were fully informed, especially about the potential disadvantages of bronchoscopic treatment (BT) compared to surgical resection [7], and they underwent treatment only after obtaining informed consent.

Inclusion criteria were accessibility of the tumor for fiberoptic bronchoscopy and high-resolution computed tomography (CT) scans showing no signs of bronchial wall infiltration (bronchial wall irregularity, bronchial wall thickening, peribronchial tumor invasion) or enlarged lymph nodes; patients with intraluminal tumors involving the main airways, ≥2 cm, volume < 5 cm^3^ and a small base attack < 1.5 cm^2^, with clinical conditions not fit for surgery (advanced age, renal insufficiency, heart failure and respiratory failure) are eligible for endoscopic treatment [8]. Exclusion and eligibility criteria for referral of patients to surgical treatment are the location of the BC (peripheral tumors) and the apparent good health of the patients (FE > 50%, FEV1 > 70%, good renal clearance, etc.) [9]. 

As premedication, puffs of lidocaine (2% to 4%) were given, nebulized through the vocal cords during a laryngoscopy. Patients were oxygenated with a balloon-valve mask with 15 L/min of 100% oxygen to promote adequate saturation for approximately 5 min before proceeding to intubation with rigid bronchoscopy. All patients underwent general anesthesia by intravenous anesthesia using: midazolam 10 mg; propofol 3 mg/kg/h; Fentanest 0.2 mg/kg/h; rocuronium 0.15 mg/kg intravenously for neuromuscular block.

After intubation, the removal of the mucoid impaction showed an endobronchial tumor originating from the airway wall of the right or left main bronchus, the rigid bronchoscope can be placed in the obstructed main bronchus, ensuring good ventilation management and avoiding blood accumulation in the contralateral bronchus. The lesions were polypoid in appearance, rounded, pinkish in color, and well vascularized, with a hard-elastic consistency [10]. 

**Figure 1:**
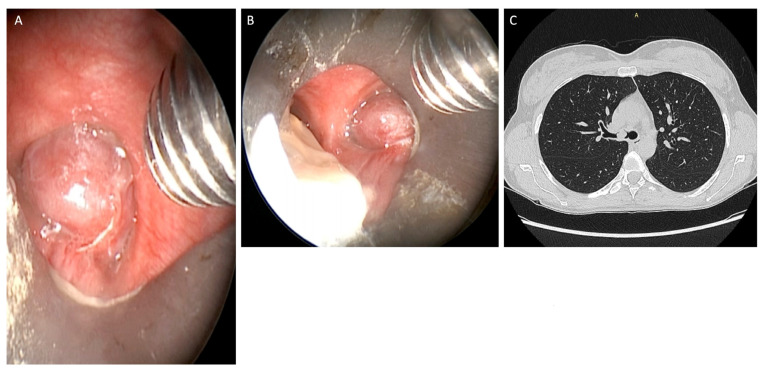
Well-vascularised polypoid lesion of 9 mm in diameter, rounded, pink in colour, with a hard elastic consistency (**A**,**B**) detected at the beginning of the right main bronchus (**C**).

They were positioned at the entrance to the right or left main bronchus just below the carina, so as to completely obstruct the airway lumen (Figure 1). Rigid and flexible bronchoscopes were used to obtain the maximum amount of tissue for correct histological diagnosis.

**Figure 2:**
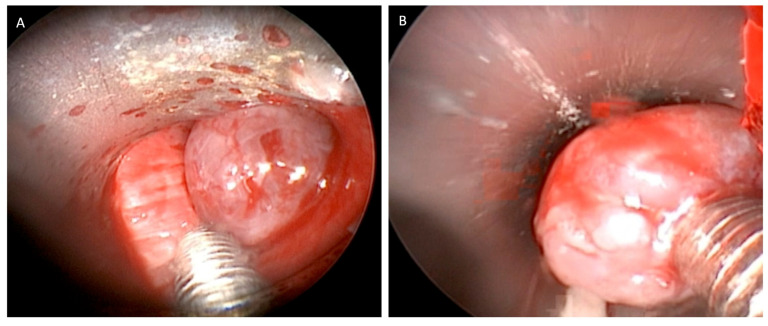
(**A**,**B**) Clearance of tumor was mostly performed by mechanical removal and through the working channel of the rigid bronchoscope of size 8.5 mm.

Hemostasis of the tumor implant base was obtained preventively using the argon plasma laser [11] or Thulep Laser (Revolix, Lisa Laser Producer OMG, Katelenburg, Germany) with high pulse-emission frequencies (up to 1000 Hz), in view of the high bleeding risk of these tumors, then tumor clearance was mostly performed by mechanical debridement and excision with the use of forceps and aspirator through the working channel of the 8.5 mm-sized rigid bronchoscope (Figure 2). 

**Figure 3:**
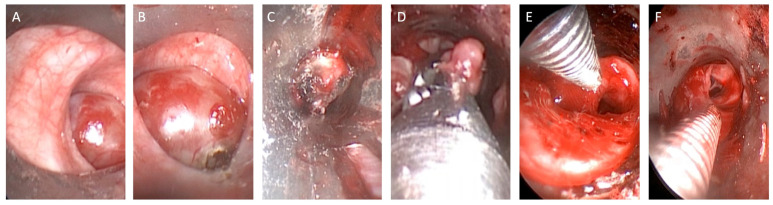
Argon plasma coagulation and diode laser (980 nm wavelength from 4 to 25 W in pulsed mode) were used to perform implant base coagulation and hemostasis during endobronchial treatment (**A**–**C**). Total lesion removal was performed using forceps and coagulation stages with automatic peak voltage control (**D**,**E**). All bronchial branches were explored after debulking (**F**).

After completing the removal of the tumor in its entirety, careful control of the hemostasis was performed with an automatic peak voltage control [12] (Figure 3).

All bronchial branches were finally explored after debulking, confirming the absence of bronchial wall thickening; however, only two patients had remarkable bleeding despite the use of cautery on the cut surface of the residual tumor. We advanced the rigid bronchoscope into the right or in the left main bronchus to prevent blood flooding into the trachea, and thus accurate hemostasis was obtained by coagulation with argon plasma or Thulep Laser (Revolix, Lisa Laser Producer OMG, Katelenburg, Germany), adrenaline (1 mL of 1 in 10,000) and SURGICEL [13]. There were no complications during the treatment. The bleeding was easily controlled without oxygen desaturation. The follow-up period was 94 months. Follow-up bronchoscopies were performed at the 1st, 3rd and 6th month and at the end of the 1st year; subsequently, after the 1st-year follow-up period, bronchoscopies were performed every 6 months [14]. High-resolution computed tomography (HRCT) was performed within 1 month after treatment, to determine extraluminal versus intraluminal tumor growth as assessed by flexible bronchoscopy. Endoscopic Treatment was considered successful when there was no sign of residual disease [15].

HRTC was then performed semiannually in the first 2 years and then annually for up to 5 years.

## 3. Results

The present study was designed as a retrospective multicentric study in which all data were retrospectively reviewed for patients with BC in the central belonging to the Thoracic Surgery Units of Luigi Vanvitelli University of Naples and Sant’Andrea Hospital in Rome. Overall, 35 patients, 13 of whom were female, were included in the study (median age, 53 years; range, 29–75 years). The median follow-up was 94 months (range, 22–95 months). A total of 26 patients (74%) had a typical carcinoid tumor. 

Immunostainings were positive for synaptophysin and chromogranin, while the Ki67 proliferation index was <5% [16]. Instead, nine patients (26%) had an atypical carcinoid tumor, despite there being no symptoms of paraneoplastic syndrome, and the patients underwent laboratory tests with a dosage of serotonin, ACTH and urinary 5-hydroxyindoleacetic acid [17], which were normal.

The prognosis mainly depends on the grade of the tumor and its anatomical extent. Staging and screening procedures were carried out by contrast-enhanced computed tomography (CT) for all study patients before any therapeutic treatment. CT showed total atelectasis of the entire right or left lung, and fiberoptic bronchoscopy showed complete obstruction of the left or right main bronchus and estimated localization, tumor size and visibility of distal tumor margin. Endobronchial treatment provided complete tumor eradication in all patients with intraluminal, pedunculated or sessile carcinoid involving the main airways, >2 cm (60% in the right main bronchus and 37% in the left main bronchus), volume < 5 cm^3^ and a small base attack < 1.5 cm^2^ (74% in typical carcinoid), Only one patient died of renal failure during the 41-month follow-up period. All patients (100%) were symptomatic. The clinical examination showed fluctuating ronchi located at the wheeze in the right and left lung. The most common signs and symptoms were: dyspnea in 31 cases (88%), cough in 33 cases (94%), chest pain in 8 cases (23%), sputum in 29 cases (83%), hemoptysis in 13 cases (37%) and other symptoms (fever, weight loss, asthenia) in 4 cases (11%). Forced expiratory volume in one second (FEV1) was reduced (range < 50%) in all patients. Overall, 21 patients had bronchial carcinoid tumors located in the right main bronchus (60%) and 13 patients had carcinoid tumors located in the left main bronchus (37%). The patient had a Glasgow Coma Scale score—13/15 (E3V4M6), arterial blood pressure—150/90 mmHg (P.A.M. 110; standard range 70 ± 110), a heart rate—133 beats/min (165 ± 110 male heart rate; 171 ± 121 female heart rate), respiratory rate—38 breaths/min (standard range in adults 16 ± 20) and oxygen saturation—86% on room air (standard range 95–98%). The arterial blood gas on room air showed a PaO_2_: 61 mmHg (standard range 73–99 mmHg), PaCO_2_: 56 mmHg (standard range ≥ 40 mmHg), HCO_3_—28.3 mmol/L (22–29 mmol/L) and pH of 7.22 (standard range 7.37–7.43) suggestive of acute hypercapnic respiratory failure (pH 7.25–7.35, PaCO_2_ media ± SD: 54.5 ± 9.6 mmHg, PaO_2_/FiO_2_ media ± SD: 134.6 ± 7.4 mmHg). The respiratory system examination revealed absent left- and right-sided air entry, and the rest of the systemic examination and laboratory investigations were unremarkable.

All patients were treated with the laser and debulking with curative intent. 

Only two patients had controlled bleeding complications; however, bleeding was well-controlled without patient desaturation. We found only two recurrences [18] in the left and right main bronchus, in patients with atypical carcinoma during fiberoptic bronchoscopy follow-up. The patient underwent bronchoscopic resection of recurrences using Thulep laser and/or argon plasma coagulation; subsequently, no other lesions were visible. The treatment of these tumors has been an essentially conservative resection.

The results are summarized in Table 1.

We compare patients undergoing endoscopic treatment with a group of 70 patients undergoing VATS lobectomy surgery for carcinoid, referred to the Thoracic Surgery Units of Luigi Vanvitelli University of Naples and Sant’Andrea Hospital in Rome between October 2012 and December 2022. The eligibility criteria for the referral of patients to surgical treatment are the peripheral site of the BC and the apparent good clinical condition of the patients (FE > 50%, FEV1 > 70%, good renal clearance, etc.). The exclusion criteria were the clinical conditions unsuitable for surgery (advanced age, renal insufficiency, heart insufficiency, respiratory insufficiency). Clinical examination showed fluctuating rhonchi in 31 patients (40%), wheezing in 19 patients (27%) and right and left hemithorax in 27 patients (39%). The most common signs and symptoms were: dyspnea in 41 cases (59%), cough in 59 cases (84%), chest pain in 11 cases (16%), sputum in 39 cases (56%), hemoptysis in 28 cases (40%) and other symptoms (fever, weight loss, asthenia) in 13 cases (19%). Forced expiratory volume in one second (FEV1) was >50% in all patients. Patients underwent pulmonary lobectomy under general anesthesia with single lung ventilation. Overall, 26 (37%) patients underwent a right upper lobectomy, 19 (27%) a right lower lobectomy, 11 (16%) a left lower lobectomy, 9 (13%) a left upper lobectomy and 5 (7%) a medium lobectomy. In total, 21 patients (30%) had an atypical carcinoid tumor. There were no deaths, and only three patients had prolonged air leaks (4%) which resolved spontaneously in 9 days. We found only one recurrence (3%) in patients who had undergone an upper left lobectomy and in patients with atypical carcinoma during the fiberoptic bronchoscopy follow-up. The patient underwent bronchoscopic resection of recurrences using a Thulep laser and/or Argon Plasma coagulation; subsequently, no other lesions were visible (Table 2).

An accurate eradication of the tumor implantation site was performed using the Argon Plasma (Erbe Argon Plasme coagulater, Marietta, GA, USA) and Thulep Laser. All patients received a follow-up of 94 months (range 22–95 months) the mean follow-up time was approximately 5 years. All patients were evaluated for recurrence by both endoscopy and chest CT surveillance. In the group of patients undergoing surgery, only one showed recurrence; recurrence was evaluated both with Tc and with endoscopic examination. In patients who underwent endoscopic surgery, the recurrence occurred along the site of the primary tumor, whereas only in the patient who underwent surgery who relapsed along the line of the bronchial suture.

### Statistical Analysis

**Figure 4:**
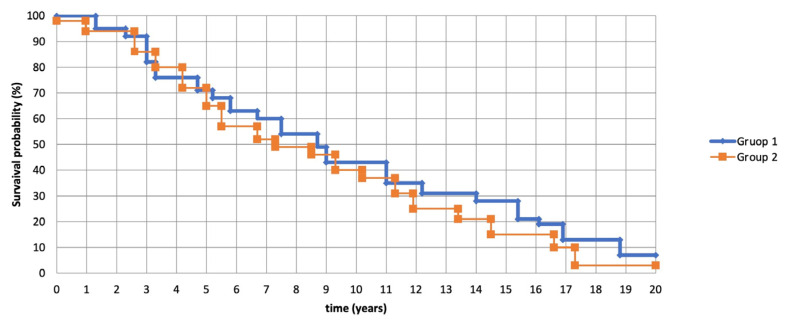
Kaplan–Meier estimator: there were no significant differences between patients who received endobronchial treatment (Group 2) and patients who received surgical treatment (Group 1) in terms of survival in the present study.

The present retrospective cohort study is based on the experience of two centers on the sample corresponding to previous studies in which selected patients with an inoperable condition. In the first analysis, there were no significant differences between patients receiving endobronchial treatment (group 2) and patients receiving surgical therapy (group 1) (Figure 4). A *p*-value measurement > 0.05, it means statistically insignificant. The statistical software MedCalc (version 12.3; Broekstraat 52, Mariakerke, Belgium) was used for the analysis [19].

## 4. Discussion

Surgery is the treatment of choice for bronchial carcinoid tumors, lobectomy or segmentectomy are preferred for peripheral tumors and sleeve resection or sleeve lobectomy is the best option for centrally located lesions [20]. However, recently, some authors have suggested that the bronchoscopic treatment effect could be a viable alternative to surgical resection in selected patients with intraluminal bronchial carcinoid [21,22]. 

Cavaliere et al. [23] described the application of the Nd:YAG laser (Laser Industries, LTD, Israel) in 38 patients with intraluminal bronchial carcinoids with a success rate of 92% with a median follow-up of 24 months free from recurrence. In our study, we applied a bronchoscopic resection with curative intent, in clinically inoperable patients affected by typical and atypical centrally located, sessile or pedunculated intraluminal carcinoids. In our experience, only two patients with atypical intraluminal carcinoid tumors experienced endobronchial recurrences during a bronchoscopic follow-up, effectively treated with endobronchial laser application, no bronchial stenosis was detected during the 51-month follow-up period [24]. 

It is difficult, clinically, to discern whether there is a residual tumor at the resection margin during endobronchial surgery. Previous studies have reported that additional laser ablation at the tumor bed was effective in preventing tumor recurrence [25,26,27]. Therefore, we performed laser and Argon Plasma Coagulation (APC) therapy after resection. 

Petrella et al. [28] believe that bronchoscopy and CT scans should be performed within 6 weeks of endoscopic treatment to evaluate the treatment outcome. In this study, the authors state that treatment success was defined as the absence of residual disease during the first 2 years of follow-up with CT and fiberoptic bronchoscopy. Therefore, it has been reported in the literature that the rates of distant locoregional recurrence were lower in patients treated with endoscopic treatment (0–5% and 0–4%, respectively), than in patients treated surgically (0–8% and 8–23%, respectively). 

Sutedja et al. [29] reported in patients undergoing Nd-YAG laser treatment no recurrences have been reported; however, some serious complications have been reported, such as perforation and fire.

Conversely, in our experience with the Argon plasma laser, Thulep Laser and subsequent tumor debulking we have found that the treatment is safer for several reasons: (1) effective and safe coagulation, even in the case of larger areas, (2) small but uniform coagulation depth and (3) reduced smoke and vapor production and controllable coagulation depth (0.5–3.0 mm) [30]. 

The cold device uses extremely low temperatures (−20 to −40 °C) to destroy endobronchial tumors by exploiting the cytotoxic effects of tissue freezing, thereby causing tissue death. Cryotherapy is a safe method, with no danger of perforation of the bronchial wall, no risk of electrical accidents or fires, no exposure to radiation. The success of the freezing injury depends on: the lowest temperature reached [31], the rate of cooling [31,32], the rate of thawing [33] and the repeated cycles of freezing and thawing [34]. The application of a low-temperature probe to a tissue induces the appearance of intra and extracellular ice crystals [35], these crystals damage the intracellular organelles, in particular the mitochondria. Although cryotherapy is a unique destructive method based on the cytotoxic effects of cold on living tissue, it is safe, less expensive and easier to use, but it is less versatile than electrocautery and has a delayed effect.

Instead, the hot device can be used to “cut” and “coagulate the tissue”. Laser light has the following properties: monochromaticity, coherence and collimation. Therefore, all light waves have the same wavelength, light waves are parallel and travel in phase with each other, light travels in only one direction, with minimal divergence. [36] The CO_2_ laser is also a “line of sight” laser, and the laser pulse travels until it is absorbed by the tissue. All medical applications of lasers refer to thermal interaction: converting the energy of optical radiation into heat resulting in the consequent coagulation or vaporization of heated tissue.

The Argon laser is mainly suitable for coagulation of blood vessels due to the strong absorption of its blue and green radiation in hemoglobin. On the other hand, the Nd:YAG laser beam, due to its low absorption and scattering, penetrates deeper into the tissue, and therefore deep tissue coagulation occurs. This type of laser can also be used for cutting, especially in cases where there is a thick area of necrosis, or for cutting blood-rich tissue, because the induced shock wave causes tissue destruction [37,38]. Laser treatment is therefore an effective method for the treatment of obstructive lesions of the airways and for tissue coagulation. The operator, however, must pay attention to avoid injury to the bronchial wall and subsequent recurrence of luminal stenosis [39].

Therefore, from an oncological point of view, we suggest that in relation to our experience bronchoscopic treatment in selected patients can also be an effective alternative as it allows a “tissue saving” which eliminates the need for surgical procedures thus improving the quality of life of patients. However, our study has limitations; it is not a study randomized controlled trial and its design is retrospective.

## 5. Conclusions

Endobronchial treatment is a parenchyma-preserving safe alternative to surgical resection in patients with intraluminally located carcinoids. Its minimally invasive nature in combination with locoregional advantages, such as recanalization of the bronchi and better evaluation of the tumor borders, certainly make it an interesting therapeutic approach for patients with intraluminal localized carcinoids; therefore, bronchoscopic resection is a valid and effective alternative for patients unsuitable for surgery.

## Figures and Tables

**Table 1:** Patients’ characteristics.

Variables	Total (*n* = 35)
Gender	
Male	22 (63%)
Female	13 (37%)
Histology	
Typical carcinoid	26 (74%)
Atypical carcinoid	9 (26%)
Localization	
Right main bronchus	21 (60%)
Left main bronchus	13 (40%)
Signs and symptoms	
Dyspnea	31 (88%)
Cough	33 (94%)
Chest pain	8 (23%)
Sputum	29 (83%)
Hemoptysis	13 (37%)
Other (fever, weight loss, asthenia)	4 (11%)
Recurrences	2 (8%)
Deaths	1 (3%)

**Table 2:** Characteristics of patients undergoing surgery.

Variables	Total (*n* = 70)
Histology	
Typical carcinoid	49 (70%)
Atypical carcinoid	21 (30%)
Type of surgery	
Right upper lobectomy	26 (37%)
Right lower lobectomy	19 (27%)
Left upper lobectomy	9 (13%)
Left lower lobectomy	11 (16%)
Medium lobectomy	5 (7%)
Clinical examination	
Fluctuating rhonchi	31 (40%)
Wheezing	19 (27%)
Right and left hemithorax	27 (39%)
Signs and symptoms	
Dyspnea	41 (59%)
Cough	59 (48%)
Chest pain	11 (16%)
Sputum	39 (56%)
Hemoptysis	28 (40%)
Other (fever, weight loss, asthenia)	13 (19%)
Recurrences	
In upper left lobectomy	1 (3%)
and atypical carcinoma	
AIRL LEAKS	3 (4%)
no Deaths	-

## Data Availability

Data are contained within the article.
